# Recurrent obstruction after surgical gastrojejunostomy in a rare case of gastric outlet obstruction: endoscopic ultrasound-guided gastrojejunostomy to the rescue

**DOI:** 10.1055/a-2197-9004

**Published:** 2023-11-20

**Authors:** Jayanta Samanta, Jahnvi Dhar, Jayendra Shukla, Anurag Sachan, Aravind Sekar, Pankaj Gupta, Antonio Facciorusso

**Affiliations:** 1 29751 Department of Gastroenterology, Post Graduate Institute of Medical Education and Research, Chandigarh, India; 2 29751 Department of Pathology, Post Graduate Institute of Medical Education and Research, Chandigarh, India; 3 29751 Department of Radiodiagnosis and Imaging, Post Graduate Institute of Medical Education and Research, Chandigarh, India; 4 18972 Department of Medical and Surgical Sciences, University of Foggia, Foggia, Italy

A 49-year-old man with alcohol-related chronic calcific pancreatitis presented with abdominal pain, vomiting, and weight loss (12 kgs) for 5 months. He had undergone truncal vagotomy with antecolic gastrojejunostomy 3 years previously for peptic ulcer disease with gastric outlet obstruction (GOO). After initially improving, he again became symptomatic with intermittent symptom-free intervals on proton pump inhibitors.

On admission, investigations revealed anemia (hemoglobin 8.7 g/dL) and hypoalbuminemia (2.9 g/dL). Esophagogastroduodenoscopy (EGD) showed confluent ulceration extending from the pylorus to the previous surgical gastrojejunostomy site ( Fig. 1 ). Histopathology from a biopsy of the ulcer bed showed eosinophilic infiltrates (40–50 per high-power field) with microabscesses, establishing the diagnosis of eosinophilic gastroenteritis (EoGE) ( Fig. 2 ). He underwent efferent loop dilation, with no improvement. A barium meal follow-through revealed long-segment strictures in both the afferent (8.5 cm) and efferent loops (22 cm), suggestive of active EoGE ( Fig. 3 ). A nasojejunal tube was placed deep into the normal part of the efferent loop of the jejunum under fluoroscopic guidance.

**Fig. 1:**
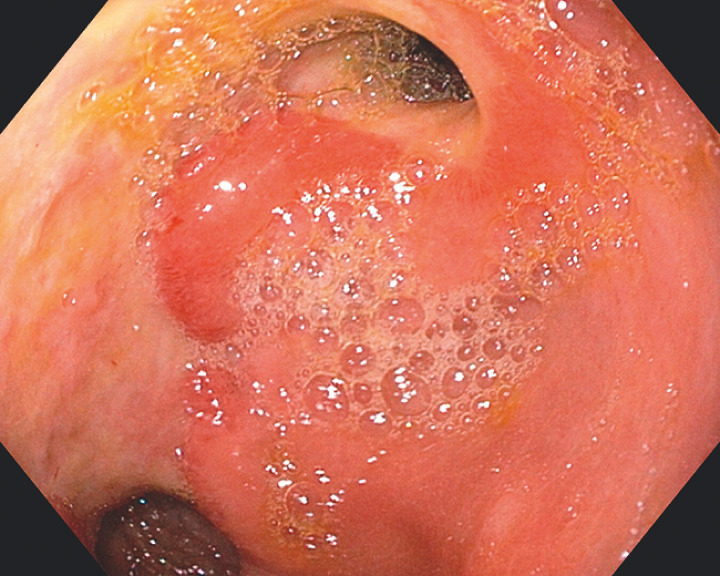
Endoscopic image showing confluent ulceration extending from the pylorus to the site of the previous surgical gastrojejunostomy.

**Fig. 2:**
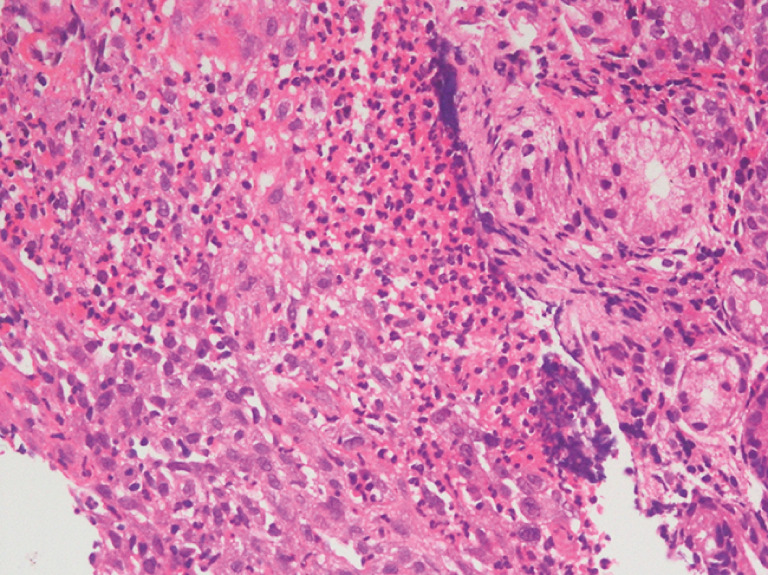
Microscopic appearance of a biopsy from the ulcer bed showing eosinophilic infiltrates (40–50 per high-power field) with microabscesses, consistent with the diagnosis of eosinophilic gastroenteritis.

**Fig. 3:**
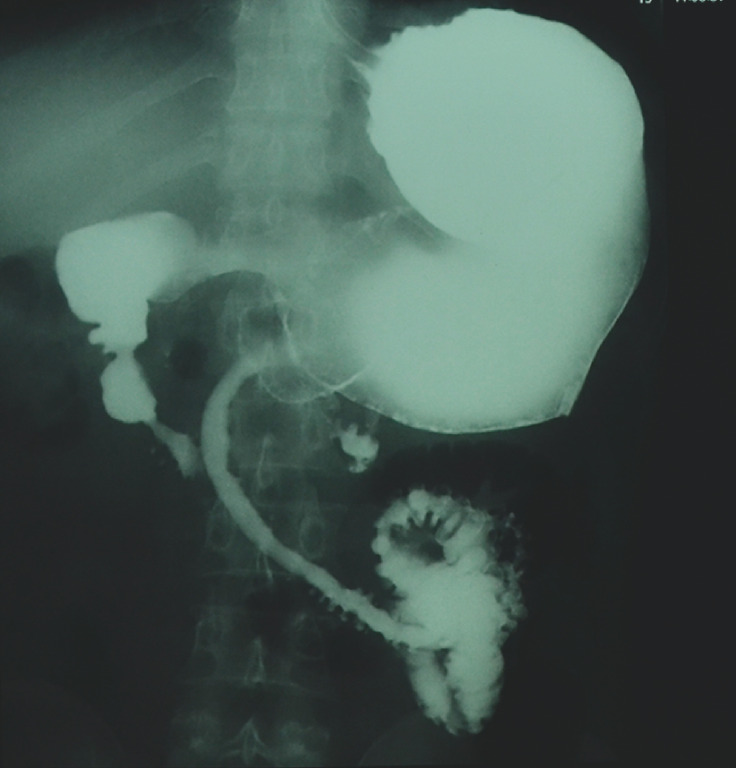
Radiographic image from the barium meal follow-through showing a long-segment stricture (22 cm) in the efferent loop, suggestive of active eosinophilic gastroenteritis.

In view of his active disease and the poor outcomes of redo surgery, the patient opted to undergo endoscopic ultrasound-guided gastrojejunostomy (EUS-GJ). Under EUS and fluoroscopic guidance, an EUS-GJ was performed using the “free-hand” approach and a 20-mm lumen-apposing metal stent (LAMS; Axios, Boston Scientific, Marlborough, Massachusetts, USA) was deployed ( Video 1 ). The trick in this case was to identify a fully distended, healthy, uninvolved segment within the efferent loop of the jejunum as the puncture site ( Fig. 4 ). Subsequently, the LAMS was balloon dilated up to 15 mm. Fig. 5 shows the two anastomosis sites: the surgical gastrojejunostomy and the EUS-GJ.

**Fig. 4:**
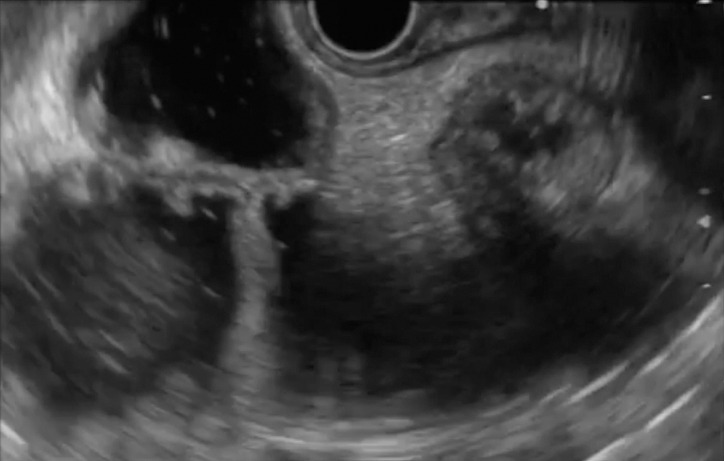
Image during endoscopic ultrasound of the jejunal loops to select a healthy, uninvolved, dilated loop for puncture to create the gastrojejunostomy, avoiding the thickened diseased loops affected by active eosinophilic gastroenteritis.

**Fig. 5:**
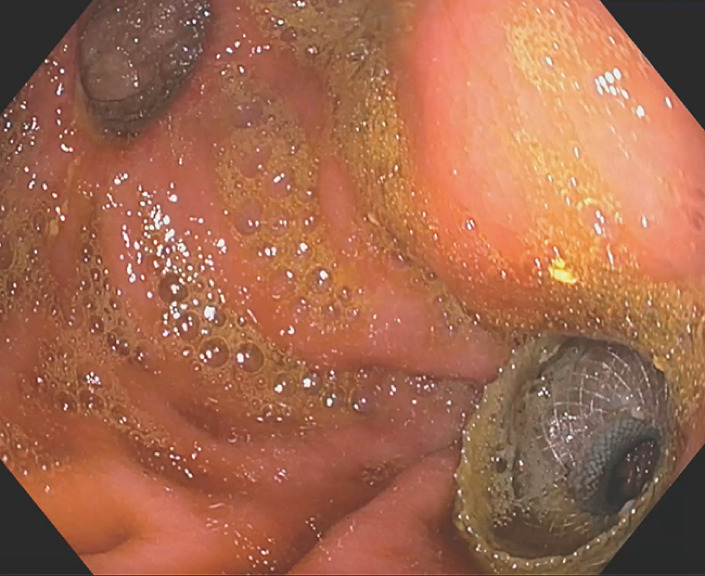
Endoscopic image showing the two anastomosis sites: the endoscopic ultrasound-guided gastrojejunostomy with the lumen-apposing metal stent visible and the pervious surgical gastrojejunostomy.

Endoscopic ultrasound-guided gastrojejunostomy using a lumen-apposing metal stent is performed for a patient with symptomatic recurrence following a prior surgical gastrojejunostomy for gastric outlet obstruction, due to active eosinophilic gastroenteritis.Video 1

The patient was discharged on steroids and elemental diet to manage his EoGE; he was asymptomatic at 1-year follow-up. It is planned that he will undergo LAMS replacement if tissue ingrowth/recurrence occurs and he remains under follow-up to ensure the active disease is controlled.

EoGE is an extremely rare but treatable cause of GOO 1 . This is the first case report of an EUS-GJ being performed as rescue therapy for recurrence following a surgical gastrojejunostomy in a patient with active EoGE; this is a technically feasible and viable option.

Endoscopy_UCTN_Code_CPL_1AL_2AB

## Notes

Endoscopy E-Videos https://eref.thieme.de/e-videos
*E-Videos* is an open access online section of the journal *Endoscopy* , reporting on interesting cases and new techniques in gastroenterological endoscopy. All papers include a high-quality video and are published with a Creative Commons CC-BY license. Endoscopy E-Videos qualify for HINARI discounts and waivers and eligibility is automatically checked during the submission process. We grant 100% waivers to articles whose corresponding authors are based in Group A countries and 50% waivers to those who are based in Group B countries as classified by Research4Life (see: https://www.research4life.org/access/eligibility/ ). This section has its own submission website at https://mc.manuscriptcentral.com/e-videos .
